# Mitochondria-Specific Accumulation of Amyloid β Induces Mitochondrial Dysfunction Leading to Apoptotic Cell Death

**DOI:** 10.1371/journal.pone.0034929

**Published:** 2012-04-13

**Authors:** Moon-Yong Cha, Sun-Ho Han, Sung Min Son, Hyun-Seok Hong, Young-Ju Choi, Jayoung Byun, Inhee Mook-Jung

**Affiliations:** 1 Department of Biochemistry and Biomedical Sciences, College of Medicine, Seoul National University, Seoul, Korea; 2 Medifron-DBT Inc., Ansan, Kyunggi-do, Korea; Thomas Jefferson University, United States of America

## Abstract

Mitochondria are best known as the essential intracellular organelles that host the homeostasis required for cellular survival, but they also have relevance in diverse disease-related conditions, including Alzheimer's disease (AD). Amyloid β (Aβ) peptide is the key molecule in AD pathogenesis, and has been highlighted in the implication of mitochondrial abnormality during the disease progress. Neuronal exposure to Aβ impairs mitochondrial dynamics and function. Furthermore, mitochondrial Aβ accumulation has been detected in the AD brain. However, the underlying mechanism of how Aβ affects mitochondrial function remains uncertain, and it is questionable whether mitochondrial Aβ accumulation followed by mitochondrial dysfunction leads directly to neuronal toxicity. This study demonstrated that an exogenous Aβ_1–42_ treatment, when applied to the hippocampal cell line of mice (specifically HT22 cells), caused a deleterious alteration in mitochondria in both morphology and function. A clathrin-mediated endocytosis blocker rescued the exogenous Aβ_1–42_-mediated mitochondrial dysfunction. Furthermore, the mitochondria-targeted accumulation of Aβ_1–42_ in HT22 cells using Aβ_1–42_ with a mitochondria-targeting sequence induced the identical morphological alteration of mitochondria as that observed in the APP/PS AD mouse model and exogenous Aβ_1–42_-treated HT22 cells. In addition, subsequent mitochondrial dysfunctions were demonstrated in the mitochondria-specific Aβ_1–42_ accumulation model, which proved indistinguishable from the mitochondrial impairment induced by exogenous Aβ_1–42_-treated HT22 cells. Finally, cellular toxicity was directly induced by mitochondria-targeted Aβ_1–42_ accumulation, which mimics the apoptosis process in exogenous Aβ_1–42_-treated HT22 cells. Taken together, these results indicate that mitochondria-targeted Aβ_1–42_ accumulation is the necessary and sufficient condition for Aβ-mediated mitochondria impairments, and leads directly to cellular death rather than along with other Aβ-mediated signaling alterations.

## Introduction

Mitochondria are of great importance in cellular metabolism, survival, differentiation and homeostasis. Mitochondria are involved not only in energy production, but also in other cellular activity, including calcium buffering, signal cascade, apoptosis induction and so forth [1]. The structure of mitochondria features a double-membrane construction involving an outer and an inner membrane. The outer membrane has voltage-dependent anionic channels that enable small molecules to be permeable. The inner membrane is the main barrier to metabolites and transporter proteins, including the TOM/TIM protein complexes that help transport protein across the inner membrane [2]. Additionally, the mitochondrial inner membrane generates an ATP energy source using a respiratory chain (electron transport chain) with complexes I through V [3].

The homeostasis of mitochondria dynamics happens through a repetitive fission and fusion process, so the size and number of mitochondria is variable according to the cellular energy demands and metabolic states. Aβ influences the fission/fusion dynamic by altering the fission/fusion-related protein levels, increasing the expression of mitochondria fission genes and decreasing that of fusion genes [4]. The increased fission gene expression prompted by Aβ alters mitochondrial morphology through fragmentation, resulting in a significantly increased number of mitochondria. The morphology and motility of the mitochondria are highly related to cytoskeletal elements, including neurofilaments and microtubule proteins [5]. Aβ (Aβ _25–35_) disturbs the axonal anterograde transport and distribution of mitochondria in mouse hippocampal neurons, which leads to synaptic degeneration [6].

The neuron is one of the cells most vulnerable to mitochondrial damage, due to its high energy demands and sensitivity to reactive oxygen species (ROS) and apoptosis. There have been numerous reports of association between mitochondrial damage and diverse neurodegenerative diseases including Parkinson's disease and AD, mediated by oxidative stress [7], [8], [9], [10]. Although amyloid plaque is the hallmark of AD, consisting of extracellular aggregation of the amyloid β peptide, the causative toxic event may occur within the cell much earlier than the appearance of senile plaque. Moreover, several lines of evidence show that Aβ is generated intracellularly in AD-related human brain regions, with the toxicity of intracellular Aβ preceding toxicity by senile plaque and neurofibrillary tangles [11]. Even in the absence of amyloid plaque, Aβ_1–42_ elicits synaptic toxicity in an amyloid plaque-independent manner [12]. Regarding its localization in intracellular compartments, Aβ has been observed in mitochondria in transgenic mice expressing human mutant amyloid precursor proteins (APPs), possibly within the membrane or a membrane-associated compartment [13], [14]. The mitochondrial Aβ distribution in the brain region is parallel to areas affected by the AD pathological progress in the murine model. Furthermore, Aβ was associated with mitochondria from AD patients compared to a cohort group [15], [16]. In an AD patient, mitochondrial destruction was reported in various brain regions [17], including the severe disruption of mitochondrial cristae, vacuole association with lipofuscin (symptomatic of mitochondrial and lysosomal damage) and increased mitochondrial DNA in pyramidal neurons within the hippocampus [5], [17].

Mitochondrial dysfunction seems to be one of the earliest signs of the AD pathological process, because mitochondrial abnormality is detectable in neurons lacking neurofibrillary tangles. Early deficits of synaptic mitochondria are detected in FAD mutant APP transgenic mice, representing Aβ accumulation within the mitochondria prior to extracellular Aβ deposition [18]. However, the direct association of mitochondrial Aβ accumulation with AD pathology is uncertain. Furthermore, proof of a direct relationship between mitochondria-specific Aβ accumulation and AD-related mitochondrial dysfunction leading to cellular toxicity remains questionable. In this study we tested whether mitochondria-targeted Aβ_1–42_ accumulation, not existing in other cellular compartments, could elicit the identical morphological alteration and dysfunction in the mitochondria of an AD mouse model and in AD-mimicking cell line conditions. Cellular toxicity caused by mitochondria-specific Aβ_1–42_ accumulation was compared to that in the cellular model, induced by extracellular Aβ_1–42_ treatments that mimicked the AD pathological environment. This study used Aβ_1–42_—the main toxic species among various Aβ peptides, containing mitochondria targeting sequence—and multiple measurements from mitochondrial functions to verify that the sole mitochondria-specific accumulation of Aβ_1–42_ is a sufficient mechanism to cause the mitochondrial dysfunction that leads to cellular toxicity and death.

## Results

### Morphological alteration of mitochondria in both in vivo and in vitro AD models

**Figure 1:**
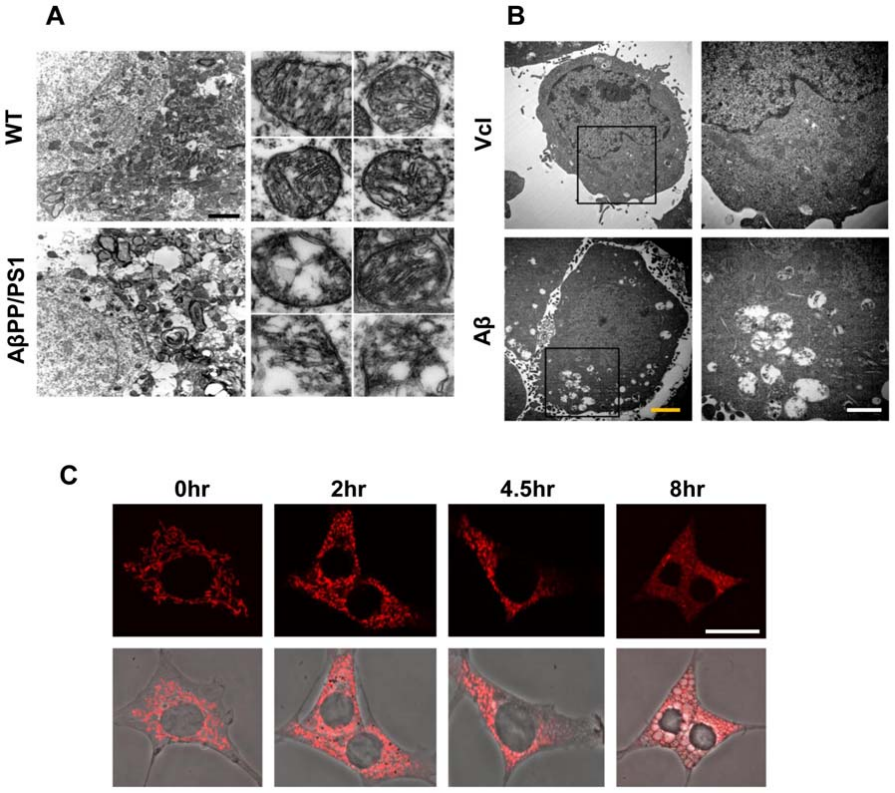
Morphological alteration of mitochondria in AβPP/PS1 mice brains and Aβ_1–42_-treated HT22 cell line. A. Electron microscopic (EM) image of mitochondria in wild type and AβPP/PS1 mice (10 months, cortex, scale bar: 2 µm) B. EM image of mitochondria in vehicle and Aβ-treated HT22 cells (yellow scale bar: 2 µm, white scale bar: 1 µm) C. Immunostaining of HSP60 in Aβ_1–42_-treated HT22 cell line by different time period of treatment (scale bar: 20 µm).

In an attempt to characterize the mitochondrial changes in both AD mice and cell line models, we investigated the morphological alteration of the mitochondria in the cortex region of AβPP/PS1 double transgenic mice (10 month) and HT22 cells treated with exogenous Aβ_1–42_. Electron microscopy (EM) revealed that the mitochondria in wild type mice showed intact, healthy structures with clear cristae and a lack of fragmentation (Fig. 1A). However, mitochondria of AβPP/PS1 double transgenic mice represented a great deal of fragmentation, vacuoles and cristae disruption—the definitive features of apoptosis. In addition, swelling drastically increased mitochondrial sizes in AβPP/PS1 double transgenic mice compared to those in wild type mice. These diverse morphological alterations of the mitochondria suggest considerable mitochondrial dysfunctions in these cells.

To examine mitochondrial deterioration by AD-like condition in cell line, HT22 cells were incubated with exogenous treatment of 5 µM Aβ_1–42_ for 7 h and analyzed using EM. The results were similar to those from previous observations of AβPP/PS1 double transgenic mice, obtained in Aβ_1–42_-treated HT22 cells, compared to those from control cells with vehicle treatment. Specifically, most of the mitochondria were swollen, exhibiting the disruption of the inner membrane and cristae in Aβ_1–42_-treated HT22 cells (Fig. 1B). For further confirmation, we performed confocal microscopy using immunofluorescence with antibodies to HSP60 (heat shock protein 60, as a mitochondrial marker) after a different time period of treatment with 5 µM Aβ_1–42_. Intact rod-shaped mitochondria started to fragmentize within 2 h of Aβ_1–42_ treatment, representing shortened forms and finally displaying a highly dispersed smear shape after 8 h of Aβ_1–42_ treatment (Fig. 1C), indicating mitochondrial fragmentation and dysfunction.

### Mitochondrial dysfunction induced by exogenous Aβ_1–42_ treatment in HT22 cells

**Figure 2:**
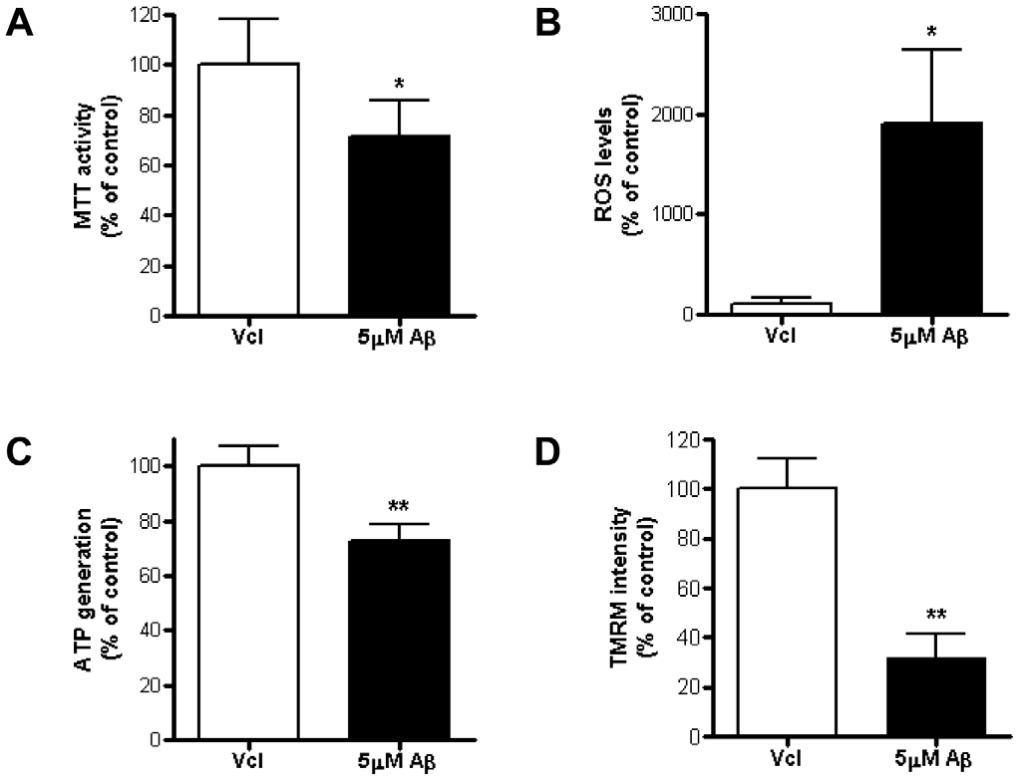
Functional assays for mitochondria in AβPP/PS1 mice brains and Aβ_1–42_-treated HT22 cell line. Four types of mitochondrial functional assays: MTT (A); ROS level (B); ATP generation (C) and TMRM intensity (D) were assessed in vehicle or 5 µM Aβ_1–42_-treated HT22 cells. MTT assay were measured after 24 h of Aβ treatment, other assays were after 6 h of Aβ treatment (* p<0.05, ** p<0.01).

Diverse functional assessments were performed to evaluate mitochondrial function under excess Aβ_1–42_ conditions. We measured four parameters: the MTT [(3-(4, 5-dimethylthiazol-2-yl)-2, 5-diphenyltetrazolium bromide)] assay for mitochondrial dehydrogenase activity; TMRM (Tetramethyl rhodamine methyl ester) intensity for mitochondrial membrane potential; fluorescent probe (CM-H_2_DCFDA) for ROS level and luciferase-based assay for ATP generation, respectively. A significant impairment of all four functional assessments occurred in Aβ_1–42_-treated HT22 cells, suggesting that the exogenous treatment of Aβ_1–42_ induces mitochondrial dysfunction (Fig. 2A–D). The presence of decreased mitochondrial dehydrogenase activity, a reduction in ATP generation, a breakdown of mitochondrial membrane potential and increased ROS generation all certainly suggest the functional impairment of mitochondria, along with morphological mitochondrial alterations (Figs. 1B and 1C) in HT22 cells by Aβ_1–42_ and the possibility that mitochondria might be the major target location for Aβ_1–42_ to accumulate and affect inside the cell.

### Endocytosis blocker inhibited Aβ_1–42_-induced mitochondrial impairments in both morphology and function

**Figure 3:**
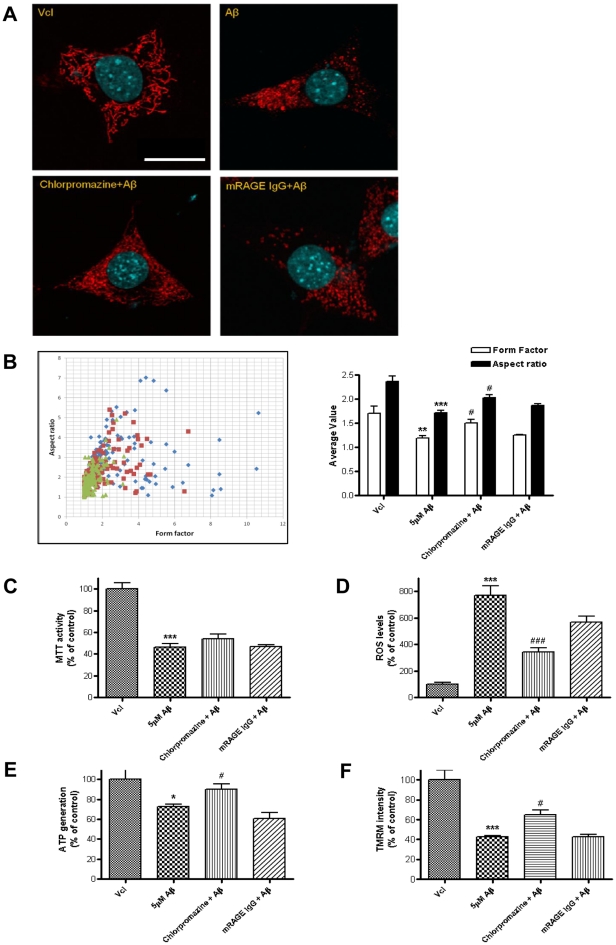
Clathrin-mediated endocytosis blocker inhibited Aβ_1–42_-induced mitochondrial dysfunction. A. Mitochondrial shapes were identified by immunostaining of HSP60 in vehicle, Aβ_1–42_, chlorpromazine+Aβ_1–42_, mouse anti-RAGE IgG+Aβ_1–42_ treatment in HT22 cells, respectively (Scale bar: 20 µm). B. Altered mitochondrial shapes are quantified using form factor and aspect ratio (blue: vehicle, red: chlorpromazine+Aβ_1–42_, green: Aβ_1–42_ in left graph). Four functional assessments of mitochondria are shown, including MTT (C), ROS levels (D), ATP generation (E) and TMRM intensity (F). * p<0.05, ** p<0.01, *** p<0.001 compared with vehicle, # p<0.05 compared with Aβ_1–42_.

Regarding the pathophysiological pathway of Aβ_1–42_, we tested whether Aβ_1–42_ needs to enter the intracellular compartment in order to elicit mitochondrial dysfunction. As Aβ_1–42_ is known to enter the intracellular compartment through clathrin-mediated endocytosis, chlorpromazine, a well-known clathrin-mediated endocytosis blocker, has been tested along with Aβ_1–42_ to investigate whether it inhibits the Aβ_1–42_-mediated alteration of the morphology and dysfunction of mitochondria. Chlorpromazine significantly inhibited the Aβ_1–42_-mediated morphological alteration of mitochondria. Elongated, thread-like HSP60 staining shapes like those seen in vehicle treatments were observed, while the co-treatment of the antagonist receptor for advanced glycation endproducts (RAGE, known receptor for Aβ_1–42_), along with Aβ_1–42_, revealed no rescuing effect at all (Fig. 3A). To quantify the mitochondrial morphological damage, we utilized a computer-based analysis that calculated form factor (FF; mitochondrial shape) and aspect ratio (AR; mitochondrial length) using the Image J program.

A quantification of the alteration of mitochondrial morphology was performed and both parameters were significantly rescued in cholopromazine+Aβ_1–42_ treatment, but not in mRAGE IgG+Aβ_1–42_ (Fig. 3B).

In addition to the morphological results, the mitochondrial functions were also rescued by a co-treatment of chlorpromazine with Aβ_1–42_ (Fig. 3C–F). Significantly increased ATP generation and mitochondria membrane potential, concomitant with significantly decreased ROS generation, were detected after cholopromazine co-treatment with Aβ_1–42_. The MTT activity was also increased by cholorpromazine co-treatment, however, not on a significant level. Taken together, the endocytosis blocker successfully inhibited Aβ_1–42_-induced mitochondrial impairments in both morphology, and function.

### Mitochondria-targeting Aβ_1–42_ caused deleterious alteration in mitochondria morphology

**Figure 4:**
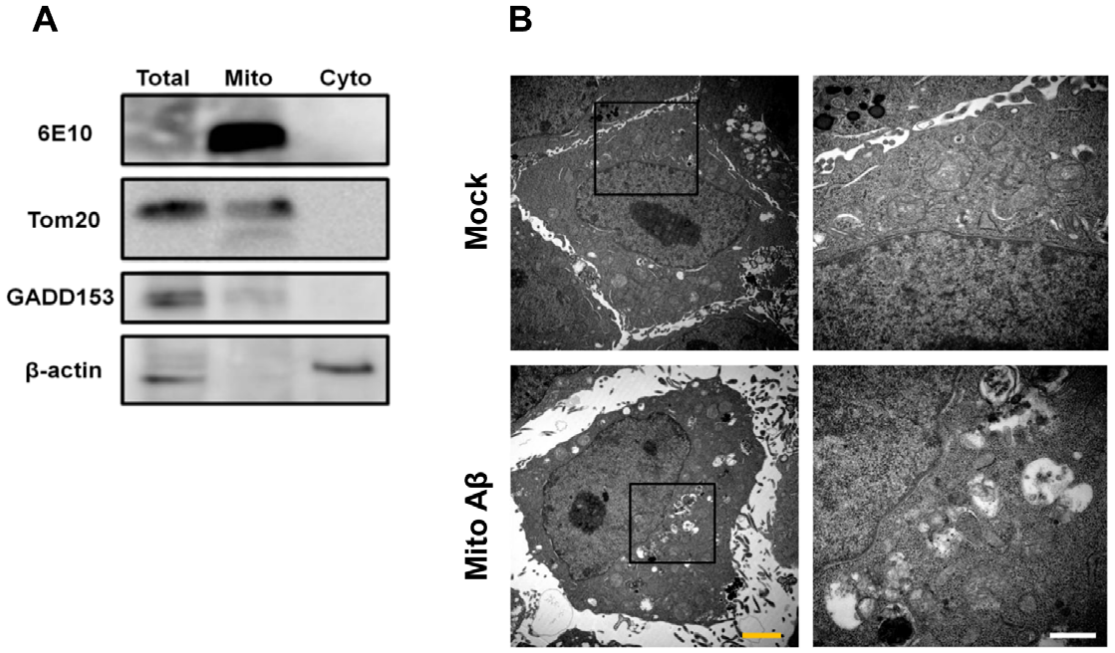
Mitochondria-specific accumulation of mito Aβ_1–42_. A. Western blot analysis showed the mitochondria-specific accumulation of Aβ_1–42_ with the presence of TOM20, mitochondrial marker and the absence of β-actin. B. EM image of mitochondria in mock and mito Aβ_1–42_-transfected HT22 cells (yellow scale bar: 2 µm, white scale bar: 1 µm).

**Figure 5:**
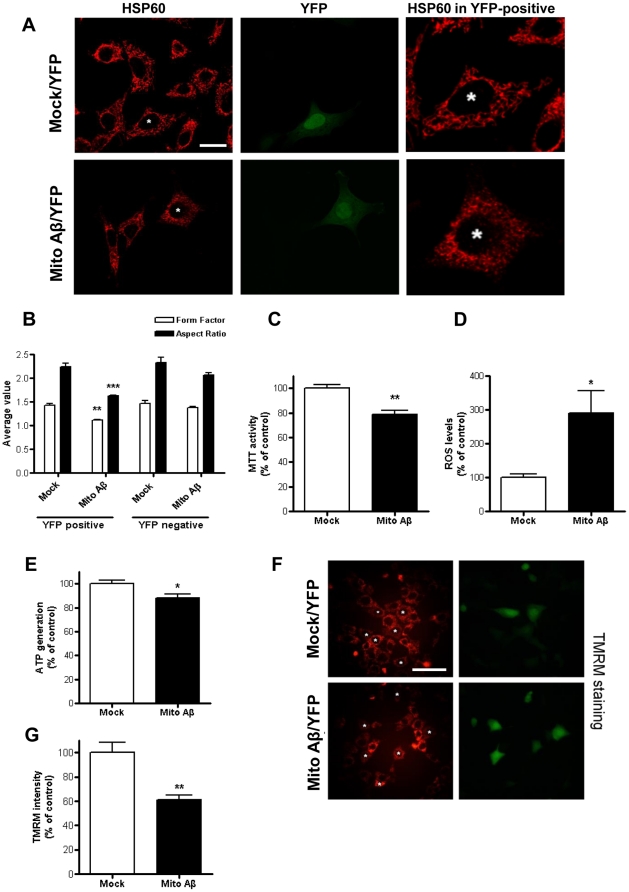
Mito Aβ_1–42_ induces not only mitochondrial morphological alteration but also functional impairments. A. Immunostaining of HSP60 and YFP in mock or mito Aβ_1–42_–transfected HT22 cells (white scale bar: 20 µm). B. Quantification of alteration in mitochondrial shape is presented as form factor and aspect ratio (** p<0.01). Four functional assessments for mitochondria are shown, including MTT (C), ROS levels (D), ATP generation (E) and TMRM staining (F). G. TMRM intensity is quantified as percent of control. * p<0.05, ** p<0.01, *** p<0.001 compared to mock, white scale bar in F: 50 µm.

As we confirmed that exogenous Aβ_1–42_ needs to enter into the intracellular compartment to elicit the deterioration of mitochondrial morphology and function, an examination was conducted to learn whether the direct accumulation of Aβ_1–42_ in mitochondria leads to morphological alteration and functional impairments in mitochondria in a way that is similar to that observed in the exogenous Aβ_1–42_ treatment. A DNA construct of Aβ_1–42_ with a mitochondria-targeting sequence (mito Aβ_1–42_) was created and transfected into HT22 cells for 12 h (Fig. S1). The specific targeting and accumulation of mito Aβ_1–42_ was observed in the mitochondrial compartment, not in cytosol, using immunoblot analysis with 6E10 (an antibody specific to the amino acid residue 1–16 of Aβ, Fig. 4A). The selective nature of subcellular fractionations was confirmed by antibodies to the compartment-selective markers: TOM20 (mitochondria specific), GADD153 (ER specific) and β-actin (cytosol specific). An EM image of mitochondria in mito Aβ_1–42_-transfected cells for 12 h showed the drastic morphological alteration of mitochondria, compared with the mitochondria in mock control, which appeared swollen and suffering from the disappearance of the inner mitochondrial membrane and invisible cristae (Fig. 4B). These morphological phenomena are similar to the mitochondrial morphology changes obtained in AD mouse models. Additionally, confocal microscopy using immunofluorescence with antibodies to HSP60 (as a mitochondrial marker) confirmed the deleterious alteration of mitochondria in mito Aβ_1–42_-transfected cells for 12 h (Fig. 5A). HSP60 staining showed shorter, less elongated and cloudy mitochondria in mito Aβ_1–42_-transfected cells, compared with the mitochondria observed in vehicle-transfected cells. The efficiency of mito Aβ_1–42_-transfection was visualized through the co-transfection of YFP, distinguishing mito Aβ_1–42_-transfected cells from non transfected cells with mito Aβ_1–42_ using green YFP staining. The quantification parameters for mitochondria shapes, FF and AR, also proved the significant deterioration of mitochondrial morphology in mito Aβ_1–42_-transfected cells, but not in both mock and non-mito Aβ_1–42_-transfected cells (Fig. 5B).

### The functional impairment of mitochondria was induced by mito Aβ_1–42_

Using the functional assays described above, we investigated whether the mitochondria-specific accumulation of Aβ_1–42_ is sufficient to cause not only morphological alterations but also functional impairment in mitochondria in mito Aβ_1–42_-transfected cells for 12 h. The mitochondria-specific accumulation of mito Aβ_1–42_ clearly impaired mitochondrial function; significantly decreasing MTT activity, decreasing ATP generation, increasing ROS generation and breaking membrane potential (Fig. 5C–F). In Figs. 5F and 5G, we showed the TMRM intensity results as well as the staining image to demonstrate that the change in mitochondrial dysfunction happened only in mito Aβ_1–42_-transfected cells (YFP positive), and not in non-transfected cells with mito Aβ_1–42_ (YFP negative).

### Apoptosis was induced by mitochondria-specific Aβ_1–42_ accumulation as well as exogenous Aβ_1–42_ treatment

**Figure 6:**
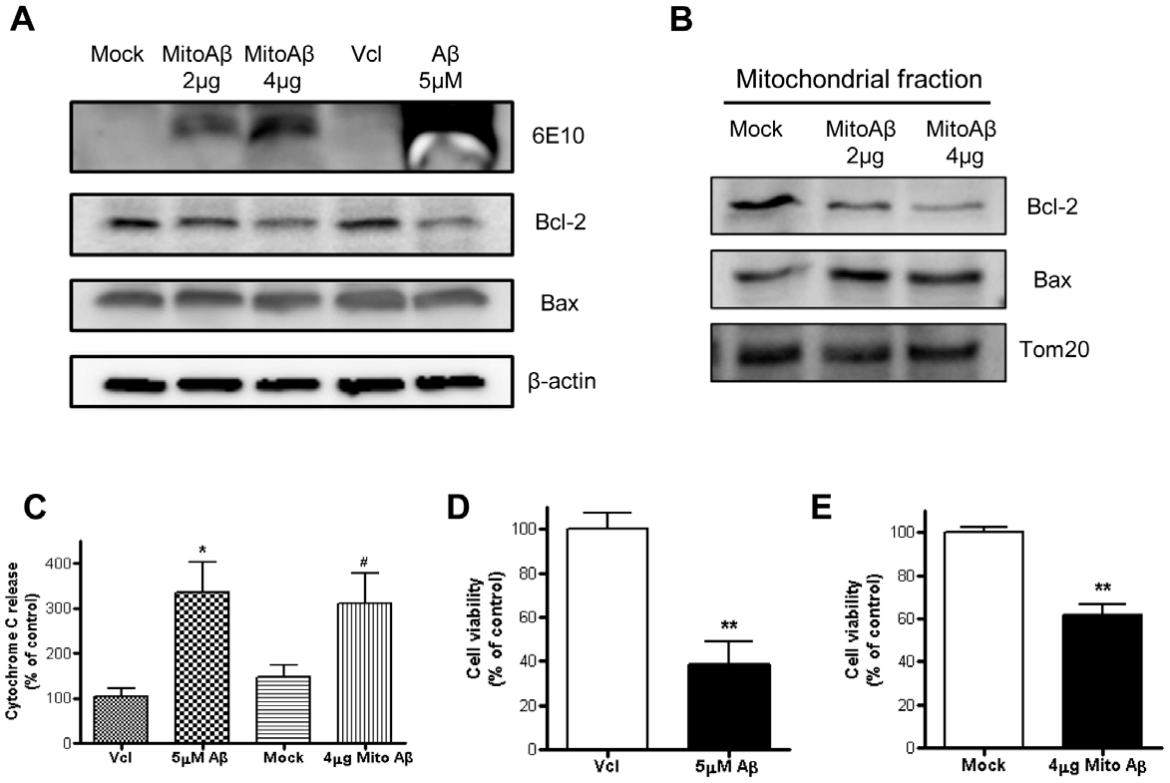
Apoptotic protein expression and cellular death in both exogenous Aβ_1–42_ treatment and mito Aβ_1–42_–transfected cells. Western blot analysis was performed in both exogenous Aβ_1–42_ treatment and mito Aβ_1–42_–transfected HT22 cells to characterize the expression level of Aβ_1–42_, Bcl-2, Bax using 6E10, Bcl-2 antibody and Bax antibody (A). Expression level of Bcl-2 and Bax was confirmed in mitochondrial fraction (B). Different concentrations of mito Aβ_1–42_ DNA constructs were used, 2 µg and 4 µg. Cytochrome C release assay (C) and calcein cell viability assay (D) were performed in vehicle-treated, exogenous Aβ_1–42_-treated, mock and mito Aβ_1–42_ transfected HT22 cells, respectively (* p<0.05, ** p<0.01 compared with vehicle, # p<0.05 compared to mock).

Morphological alteration and functional impairments of mitochondria were demonstrated as distinctive phenomena in both exogenous Aβ_1–42_ treatment and the mitochondria-specific accumulation of Aβ_1–42_ in HT22 cells. This discovery was followed by an investigation into whether these mitochondrial destructions could lead to cellular death through an apoptotic event. We measured expression level of Bcl-2 and Bax, which is widely used as the marker to initiate the apoptosis pathway by triggering cytochrome C release from mitochondria using Western blotting. When total cell lysates were examined, expression of the anti-apoptotic protein Bcl-2, decreased significantly during the mitochondria-specific accumulation of Aβ_1–42_ for 24 h, and during the exogenous Aβ_1–42_ treatment of HT22 cells while the Bax expression showed no sign of change in both environments, which implies that both situations trigger the apoptosis pathway in the same way (Fig. 6A). A higher mito Aβ_1–42_ expression (4 µg vs 2 µg of mito Aβ_1–42_ transfection) decreased Bcl-2 level, suggesting that an increased accumulation of mito Aβ_1–42_ down-regulated Bcl-2. Expression level of Bcl-2 and Bax were assessed by Western blotting using mitochondrial fraction to confirm previous data. More dramatic decreased Bcl-2 level and increased Bax level were observed in condition of mito Aβ_1–42_ expression (Fig. 6B). We also measured the release of cytochorome C from mitochondria, the subsequent event of increased Bax/Bcl-2 in the apoptosis pathway, and detected a significant increase of cytochrome C release during the mitochondria-specific accumulation of Aβ_1–42_ for 24 h, as well as during the exogenous Aβ_1–42_ treatment, compared to vehicle treatment in HT22 cells (Fig. 6C). Finally, a cell viability test confirmed that the mitochondria-specific accumulation of Aβ_1–42_ for 24 h is a sufficient event to induce cellular death, just as it is in exogenous Aβ_1–42_ treatment (Fig. 6D and 6E).

## Discussion

Aβ has attracted a great deal of interest in the field of AD research, and its influence at the cellular level, and within the neuronal system, has been the pressing issue to clarify for those with a goal of diagnostic and therapeutic development. Recent studies have highlighted the mitochondrial damage induced by Aβ under AD pathological conditions [4], [5], [7], which raises questions about the delivery mechanism of Aβ-inflicted damage to mitochondria. It is also vital that we determine whether mitochondrial impairment by Aβ is fatal enough to cause neuronal death in AD conditions.

One possible cause for mitochondrial dysfunction in AD is the amyloid precursor protein (APP) [5], [19], [20]. In both in vitro and in vivo studies, using APP-expressing HCN-1A cells and an AD SW/APP (Tg2576) mouse model, respectively, the accumulation of APP in mitochondria by mitochondrial targeting and the subsequent transmembrane arrest resulted in mitochondrial dysfunction. This in turn prompted a decline in the cytochrome c oxidase activity, reduced respiration-coupled ATP synthesis and disruption of mitochondrial membrane potential [20]. In this study, which explored mitochondrial dysfunction by transmembrane-arrested APP studies, APP with intact COOH-terminal was used and arrested as the form of APP observed in the inner mitochondrial membrane—a finding that precluded Aβ production. APP was reported to form a ∼480 kDa complex with the translocase of the outer mitochondrial membrane 40 (TOMM40) import channel in addition to a supercomplex of approximately 620 kDa with both TOMM40 and the translocase of the inner mitochondrial membrane 23 (TIMM23) import channel, which are the mechanisms for mitochondrial dysfunction by APP in AD [21]. However, the mitochondrial influence of Aβ_1–42_ accumulation in mitochondria might be different from the mitochondrial dysfunction caused by APP through the APP/TOMM40/TIMM23 complex. Yet, it is unclear whether the mitochondrial-specific accumulation of physiologically relevant Aβ_1–42_ has a similar impact on mitochondrial dysfunction. One certainty is that Aβ must enter into the cellular compartment to elicit mitochondrial dysfunction, because either the increased production of extracellular Aβ peptide or Aβ plaques failed to induce mitochondrial dysfunction [20], [22]. Therefore, mitochondrial accumulation was regarded as an essential step for Aβ-mediated mitochondrial dysfunction and several reports suggest that mitochondria are a direct location for Aβ accumulation in AD neurons, proving functional impairments of mitochondria, such as free radical generation and oxidative damage [13], [14]. Questions remain regarding whether the mitochondrial accumulation of Aβ is a sufficient event to elicit mitochondrial dysfunction or neuronal death, because mitochondrial Aβ accumulation is not the sole event resulting from the existence of excess Aβ in AD conditions. Other events may occur simultaneously in the presence of Aβ, including the activation of a signaling pathway through receptor engagement or the destruction of other intracellular organelles upon cellular entry that may trigger mitochondrial dysfunction indirectly. Also ionic dyshomeostasis, such as Ca^2+^ and Zn^2+^, may synergistically impair mitochondria via pathogenic loop [23].

Our results clearly showed that exogenously treated Aβ_1–42_ enters the intracellular compartment through clathrin-mediated endocytosis, and mitochondria-specific Aβ_1–42_ accumulation was a sufficient event to induce not only mitochondrial dysfunction, but also neuronal death. The receptor for advanced glycation endproduct (RAGE) is the binding receptor for Aβ, and RAGE-Aβ engagement has been reported to modulate diverse signaling pathways, such as NF-κB, p38 mitogen-activated protein kinase and ERK1/2 activation [24], [25], [26], [27]. Additionally, RAGE was reported to mediate the intraneuronal transport of Aβ and the consequent neuronal dysfunction [28]. However, as our Figures 3 and 4 show, blocking RAGE using anti-RAGE IgG failed to rescue the Aβ_1–42_-mediated mitochondrial disruption in both morphology and function, suggesting that RAGE-Aβ engagement is not involved in the process of mitochondrial disruption and dysfunction by Aβ. It is possible that the RAGE expression level in this HT22 cell line might to be too low to mediate the intraneuronal transport of Aβ.

Even though the majority of the mitochondrial dysfunction induced by the exogenous treatment of Aβ_1–42_ (ROS level, ATP generation and TMRM intensity) was rescued when chlorpromazine, a clathrin-mediated endocytosis blocker, was co-treated with Aβ_1–42_, the action failed to fully recover the reduced MTT activity (Fig. 3C). The co-treatment of chlorpromazine with Aβ_1–42_ produced a mild recovery of MTT activity, but not at a significant level. The MTT assay measures the cellular activity and viability within mitochondrial reductase activity. This reductase enzyme is regulated by many diverse factors because it is very relevant to the metabolic activity of cells [29]. In addition, disturbing or activating specific signaling pathways, such as ErbB2, was reported to affect the mitochondrial reductase activity, which would change the MTT result [30]. Reduced MTT was induced by both the intracellular entrance of Aβ_1–42_ followed by mitochondrial Aβ_1–42_ accumulations and the Aβ_1–42_-mediated alteration of the signaling pathway. Extracellular Aβ can facilitate AMPA receptor internalization and NMDA receptor trafficking that may destabilize intracellular Ca2+ signaling. Also, Aβ oligomers can form Ca2+-permeable pores in the plasma membrane and bind to other cell surface receptors (for example, LRP, FPRL1, and α7nAChR). These altered Ca^2+^ signaling induced by extracellular Aβ can impair intracellular organelles including mitochondria [31], [32], [33]. Moreover, even though clathrin-mediated endocytosis serves as internalization system for trafficking Aβ, clathrin-independent endocytosis can be another possible pathway for Aβ internalization [34]. For these reasons, blocking the endocytosis of Aβ_1–42_ seems to rescue certain portion of the mitochondrial reductase activity.

The mitochondria-specific targeting of Aβ_1–42_ was confirmed in two ways. The Western blot analysis shown in Figure 4 displays specific Aβ immunoreactivity only in the mitochondria fraction with the presence of Tom20 protein (mitochondrial marker) and the absence of either GADD153 (ER marker), or β-actin. Mitochondrial morphology alteration and dysfunction only occurred in YFP-positive cells, not in YFP-negative cells (Figs. 5A, 5B and 5F), which suggests that only successfully transfected mito Aβ_1–42_ cells experienced mitochondrial Aβ_1–42_ accumulation followed by the morphological alteration and dysfunction of the mitochondria.

Even though diverse variations in the altered expression level of Bax and Bcl-2 were observed in the apoptosis pathway, including increases in both [35], a decrease in Bcl-2 and an increase in Bax and a decrease in Bcl-2 alone [36], [37], the typical apoptotic pathway through CD95/Fas [38] displayed an increased Bax/Bcl-2 ratio and followed cytochrome C release from the mitochondria. Our results clearly show that the increased Bax and decreased Bcl-2 level followed by cytochrome C release from the mitochondria was mediated not only by the exogenous treatment of Aβ_1–42_, but also by the mitochondria-specific accumulation of Aβ_1–42_. These events ultimately lead to cell death, measured by the calcein viability test (Fig. 6D and 6E). Calcein-AM could be taken up by living cells and metabolized by intracellular esterases into the membrane-impermeable fluorescent calcein that disperses throughout the entire cytoplasm of the cell, and it is the intensity of this intracellular fluorescent calcein that indicates cellular viability. The involvement of CD95/Fas has not been examined in this study, however, a CD95/Fas-mediated pathway would not be excluded from future studies as a candidate for a signaling pathway for mito Aβ_1–42_-mediated cellular death. The existence of additional influences on mitochondria during the mitochondria-specific accumulation of Aβ_1–42_ using a mito Aβ_1–42_ DNA construct is probable, including mitochondrial anterograde/retrograde transport within the neuron, fission/fusion protein levels and other signaling pathways that will need to be assessed in future studies [39], [40].

## Materials and Methods

### Animals and cell cultures

AβPP/PS1 double transgenic mice (AβPP Swedish mutant and PS1 deletion exon 9 mutant; B6C3Tg (AβPPswe, PSEN1dE9)85Dbo/J; stock number 004462) were purchased from Jackson lab (BarHarbor, ME) and confirmed by genotyping. The experimental protocol was approved by the Ethics Review Committee for Animal Experimentation in Seoul National University. HT22 cells, mouse hippocampal neuronal cell line (gifted from Dr. David Schubert, Salk Institute), were cultured in Dulbecco's modified Eagle's medium (DMEM; HyClone, Irvine,CA, USA) supplemented with 10% fetal bovine serum (HyClone), 0.1 mg/ml penicillin and streptomycin (Sigma, StLouis, MO) at 37°C in a 5% CO2 incubator.

### DNA construct

Created mito Aβ sequence were based on pcDNA 3.1/hygro mammalian expression vector (Invitrogen) and full length APP cDNA using two sets of primer; 5′- AA GGTACCATGTCCGTCCTGACGCCG -3′ (mitochondria-targeting sequence), 5′-AACTC GAGCTACGCTATGACAACACCGCC C -3′ (Aβ_1–42_). Procedure is shown in Figure S1.

### Transfection

Transfection of the HT22 cells was performed using Lipofectamine LTX according to the manufacturer's protocol (Invitrogen, Carlsbad, CA). Briefly, cells were seeded in 96 well plate for the mitochondrial functional assay and in 6 well plate for fractionation of mitochondria. The mito Aβ_1–42_ construct was mixed with Lipofectamine LTX in OptiMEM (Invitrogen Carlsbad, CA). After 30 min of incubation, the mixture was added to the cell culture medium.

### Electron microscope analysis

The brain tissues of AβPP/PS1 double transgenic mice and HT22 cells were fixed overnight in a mixture of cold 2.5% glutaraldehyde in 0.1 M phosphate buffer (pH 7.2), and 2% paraformaldehyde in a 0.1 M phosphate or cacodylate buffer (pH 7.2) and finally embedded using only epoxy resin. The epoxy resin-mixed samples were loaded into capsules and polymerized at 38°C for 12 h and 60°C for 48 hours. Thin sections were made using an ultramicrotome (RMC MT-XL ; RMC Products, Tucson, AZ, USA) and collected on a copper grid. Appropriate areas for thin sectioning were cut at 65 nm and stained with saturated 4% uranyl acetate and 4% lead citrate before examination with a transmission electron microscope (JEM-1400; Japan) at 80 Kv.

### MTT assay

The medium was aspirated from plates, and 50 µl per well 2.5 mg/ml MTT in phenol red–free medium was added. Plates were incubated for 2 h at 37°C, followed by the aspiration of the MTT solution, the addition of 140 µl per well isopropanol to dissolve formazan crystals, and incubation at 37°C for 30 min. After incubation, plates were equilibrated to room temperature (RT) for an additional 20–30 min. Absorbance was measured at 540 nm.

### ROS measurement

Levels of hydrogen peroxide were determined using dichloro-fluorescein diacetate (DCFDA; Invitrogen, Carlsbad, CA). Treated cells were incubated with 1 µM DCFDA for 30 min and washed with PBS. Fluorescent signals were captured using a fluorescence microscope (Olympus, Tokyo, Japan).

### Assay for cellular ATP levels

The medium was removed from plates and washed three times with 50 µl per well PBS. 50 µl per well 1% Triton X-100 (Sigma, St Louis, MO) was added to lysed cells. 90 µl per well ATP Determinant Kit reaction solution (Molecular Probes) was added to 10 µl per well of cell lysate. Plates were agitated for 2 min and luminescence was measured.

### TMRM assay for mitochondrial membrane potential

In depolarized cells, the TMRM dye accumulates in the mitochondria. Red fluorescence serves as the indicator for mitochondrial membrane potential. The medium was removed from plates, and 100 µl per well for 500 nM TMRM (Invitrogen, Carlsbad, CA) in phenol red–free medium was added. Plates were incubated for 1 h at 37°C and washed three times with 50 µl per well PBS. Fluorescent signals were captured using a fluorescence microscope (Olympus, Tokyo, Japan).

### Fractionation of mitochondria

To isolate cytosol and mitochondrial fractions, cells were lysed by Dounce homogenizer in the buffer containing 20 mM HEPES, pH 7.5, 250 mM sucrose, 20 mM KCl, 1.5 mM MgCl_2_, 1 mM EDTA, 1 mM dithiothreitol, 1 mM phenylmethylsulfonyl fluoride, 0.2 trypsin inhibitory units/ml aprotinin, and 20 µg/ml leupeptin at 4°C. The cell lysates were centrifuged at 600×g for 10 min to remove unbroken cells and nuclei. The supernatants were centrifuged at 7000×g for 10 min at 4°C and the resulting pellet was collected as the mitochondrial fraction.

### Immunostaining

Cells were fixed for 20 min with 4% paraformaldehyde/PBS and permeabilized with 0.3% Triton X-100 for 10 min. After blocking, cells were incubated with primary antibodies (anti-Hsp60 monoclonal antibody). After PBS washing, cells were incubated for 1 h at room temperature with fluorescent-linked secondary antibodies (1∶500; Jackson Labs, West Chester, PA, USA). Cells were counterstained for 5 min with DAPI/PBS solution. The labeled cells were mounted on glass slides, and then visualized and photographed by a Confocal microscope (Olympus FV10i; Olympus, Tokyo, Japan).

### Western blotting

For whole cell lysates, cells were washed twice with cold PBS and directly lysed with 1× sample buffer. For mitochondrial fractions, cells were fractionated as described previously (Narendra et al., 2008), and mitochondrial pellets were lysed with 1× sample buffer. 20 µg proteins were separated on 10% Tris-glycine or 10% Tris-tricine SDS-PAGE. Membranes were incubated with antibodies against the indicated proteins. Enhanced chemiluminescence (ECL; GE Healthcare Biosciences, Pittsburgh, PA) was used for visualization of the signal. The images were captured using a bioimaging analyzer (LAS-3000; Fuji, Tokyo, Japan) and analyzed using a Multi-Gauge program (Fuji, Tokyo, Japan). The following antibodies were used in the study: anti-Tom20 (Santa Cruz Biotechnology, Inc.), and anti-β-actin (Sigma-Aldrich).

### Cytochrome C release

Cytosolic cytochrome C was measured using Cytochorme C ELISA kit (Invitrogen, Carlsbad, CA) according to the manufacturer's protocol. Briefly, 10 mg/ml of each cytosolic fraction was added to the well of a microtiter plat pre-coated with monoclonal anti-cytochrome C antibody, followed by adding 100 µl of cytochrome C biotin conjugate solution for 1 h at RT. 100 ul of streptavidin-HRP working solution was added for 30 min at RT. After 30 min of rocking at RT, 100 ul of stabilized chromogen was added for 30 min at RT. After 30 min, an equal volume of STOP solution was added and product was measured on a spectrophotometer at 450 nm.

### Calcein viability assay

The medium was removed from plates, and 50 µl per well 1 µM calcein-AM (Molecular Probes) in phenol red–free medium was added. Plates were incubated for 1 h at 37°C and washed three times with 50 µl per well PBS. Fluorescence was measured at excitation and emission wavelengths (ex/em) of 485 nm/530 nm.

### Image analyses

Quantitative analysis of cell counting was performed with Image Pro Plus 5.1 software (Media Cybernetics, Silver Spring, MD). For morphological analyses of mitochondria, acquired images were analyzed as described by using IMAGE J (National Institutes of Health) and Photoshop (Adobe, San Jose, CA).

### Statistical analysis

All data was expressed as means ± SEM. Student t-test was used for two-group comparisons, and analysis of variance, followed by Fisher's LSD post hoc test, which was used to compare three or more groups using SigmaStat for Windows Version 3.10 (Systat Software, Inc., Point Richmond,CA). Significance was designated as P-value<0.05

## Supporting Information

Figure S1 **Generation of mito Aβ_1–42_ construct.**
(TIF)
