# Supplementary figures and images for: Mitochondria-Specific Accumulation of Amyloid β Induces Mitochondrial Dysfunction Leading to Apoptotic Cell Death
Source: PLoS One. 2012 Apr 13;7(4):e34929. doi: 10.1371/journal.pone.0034929 (PMC3325919; doi:10.1371/journal.pone.0034929)

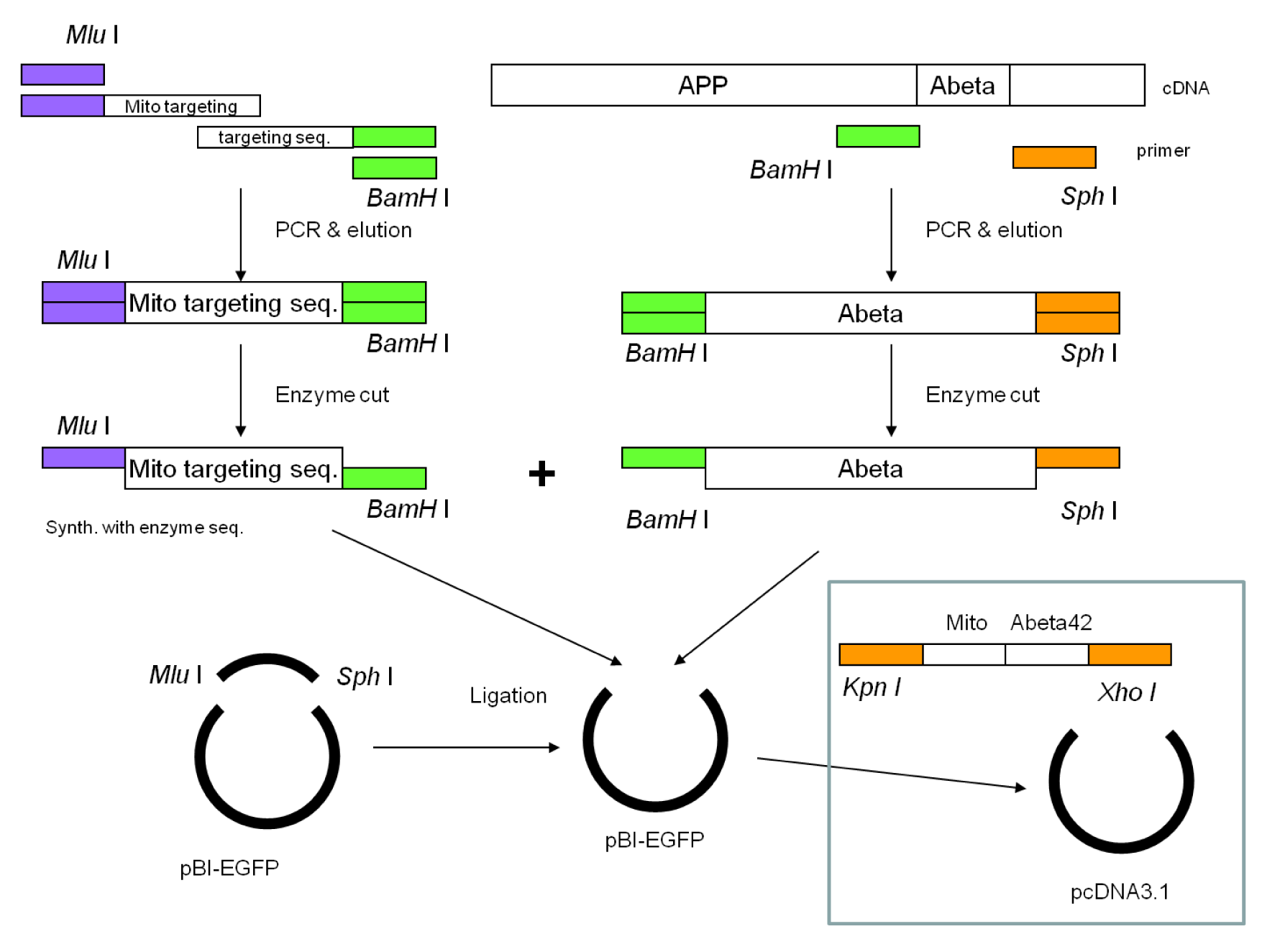

Supplement: Figure S1 — Generation of mito Aβ1–42 construct. (TIF) [file pone.0034929.s001.tif]
